# Noise Removal in the Developing Process of Digital Negatives

**DOI:** 10.3390/s20030902

**Published:** 2020-02-07

**Authors:** Marek Szczepański, Filip Giemza

**Affiliations:** Faculty of Automatic Control, Electronics and Computer Science, Silesian University of Technology, Akademicka 16, 44-100 Gliwice, Poland

**Keywords:** demosaicing, interpolation, color filter array, Bayer filter, image filtering, image noise

## Abstract

Most modern color digital cameras are equipped with a single image sensor with a color filter array (CFA). One of the most important stages of preprocessing is noise reduction. Most research related to this topic ignores the problem associated with the actual color image acquisition process and assumes that we are processing the image in the sRGB space. In the presented paper, the real process of developing raw images obtained from the CFA sensor was analyzed. As part of the work, a diverse database of test images in the form of a digital negative and its reference version was prepared. The main problem posed in the work was the location of the denoising and demosaicing algorithms in the entire raw image processing pipeline. For this purpose, all stages of processing the digital negative are reproduced. The process of noise generation in the image sensors was also simulated, parameterizing it with ISO sensitivity for a specific CMOS sensor. In this work, we tested commonly used algorithms based on the idea of non-local means, such as NLM or BM3D, in combination with various techniques of interpolation of CFA sensor data. Our experiments have shown that the use of noise reduction methods directly on the raw sensor data, improves the final result only in the case of highly disturbed images, which corresponds to the process of image acquisition in difficult lighting conditions.

## 1. Introduction

**Figure 1:**
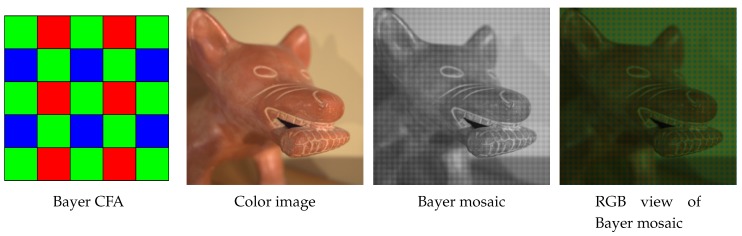
Bayer color filter array (CFA) pattern.

The vast majority of today’s digital cameras use a single sensor with a color filter array (CFA). It is therefore necessary to interpolate the missing pixels using so-called demosaicing algorithms to obtain a complete RGB image. For the color filter array, the Bayer pattern remains the most popular one [1], and most of the demosaicing techniques are designed to interpolate such data. Figure 1 shows the arrangement of color filters in a Bayer mosaic, the image captured by the CFA sensor, and its RGB visualization.

Design of efficient demosaicing algorithms is important because almost all modern digital color cameras use an image demosaicing algorithm to produce color images. Although, in the case of modern image sensors with very high resolution, even the simplest methods can give satisfactory results, the researchers all over the world continually create new improved demosaicing algorithms starting from the simplest bilinear interpolation to very sophisticated ones such as iterative color stencils technique presented by Getreuer [2] and techniques derived from machine learning presented by Khashabi et al. [3].

Unfortunately, most interpolation algorithms are not immune to image sensor noise. Interpolation errors are particularly evident in the presence of impulsive noise: it causes the formation of unpleasant and often difficult to remove artifacts in the final image. Fortunately, impulsive noise in modern imaging sensors is quite easy to remove at an early stage of image processing. The interpolation process, however, changes the characteristics of other noise generated in the image sensor, which can significantly hinder its later removal with standard filtering methods designed for color images.

The problem of noise in the imaging sensors over the years were dealt in different ways. The simplest one is to use some standard denoising methods such as VMF (Vector Median Filter) and its modifications, NLM (Non-Local Means), BM3D (Block-Matching and 3D Filtering) etc. [4,5,6,7,8,9]. Another option is to adjust noise reduction algorithms to work with raw data and remove noise before interpolating CFA data [10,11,12,13]. Another approach is to combine noise reduction and interpolation into one process—joint denoising and demosaising [3,14,15].

The standard filtering quality assessment procedure assumes the use of a set of sRGB test images with 8-bit color depth for each channel. These images are distorted by simulated noise, the distribution of which usually does not fully correspond to the processes occurring in the real imaging sensors. When demosaicing algorithms or the entire CFA image processing pipeline are tested, the test sRGB images are converted to a simulated Bayer image.

The real problem is slightly different, the data from the sensor are not in the sRGB space, but in the linear space of the sensor, usually with 12 or 14 bit depth. The noise associated with the image acquisition process should therefore be simulated directly for raw sensor data, i.e., where it occurs. When processing digital negatives, this noise is subject to a number of nonlinear transformations, as a result of which the noise in the final image may have a significantly altered distribution. This could adversely affect the performance of various filtering techniques, especially if some of them such as NLM or BM3D were designed for a specific noise model (most often Gaussian). Moreover, many commonly used test images have been obtained using CFA sensors and may contain interpolation artifacts, and often contain significant noise. These images also usually have a limited bit depth compared to real raw sensor data. It seems, therefore, that such data should not be used either to test the entire process of developing digital negatives, or to test its individual stages. Therefore, a new test image database has been prepared with high-quality reference images, raw, and disturbed raw images. Given the noise model described above, it can be assumed that the filtering process should also be carried out on raw data and not, as in the traditional approach for sRGB images at the end of the digital image development process.

In this article, we try to verify the correctness of this assumption, so we want to answer the question about the proper sequence of operations performed during the development of digital negatives, and more specifically, about the proper placement of the interpolation and filtering processes. As part of the experiment, tests were carried out for various demosaicing and filtering methods, applied in different order. During the research, the effectiveness of the examined solutions was tested using objective quality measures, and the statistical significance of the results obtained was assessed. The results of our tests can help in choosing the most effective approach to developing raw images used directly in digital cameras as well as in postprocessing software.

The paper is organized as follows. Section 2 presents general digital negatives processing pipelines and their potential impact on the final results; it also explains problems related to the objective evaluation of the quality of developed raw images. Section 3 describes how to prepare test data, ground truth images, noisy images, and images for different processing steps. Section 4 describes an experiment to assess the impact of placing noise filtering at different stages of raw image processing pipeline. Section 5 concludes our results.

## 2. Raw Image Processing Pipelines

**Figure 2:**
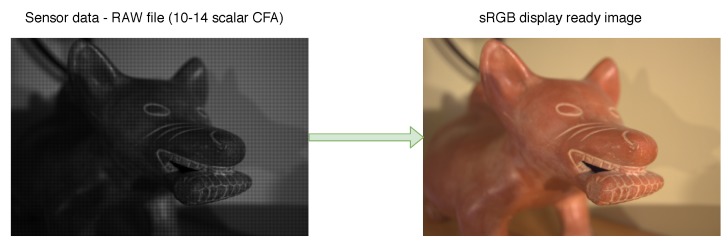
Problem of processing raw image data.

**Figure 3:**
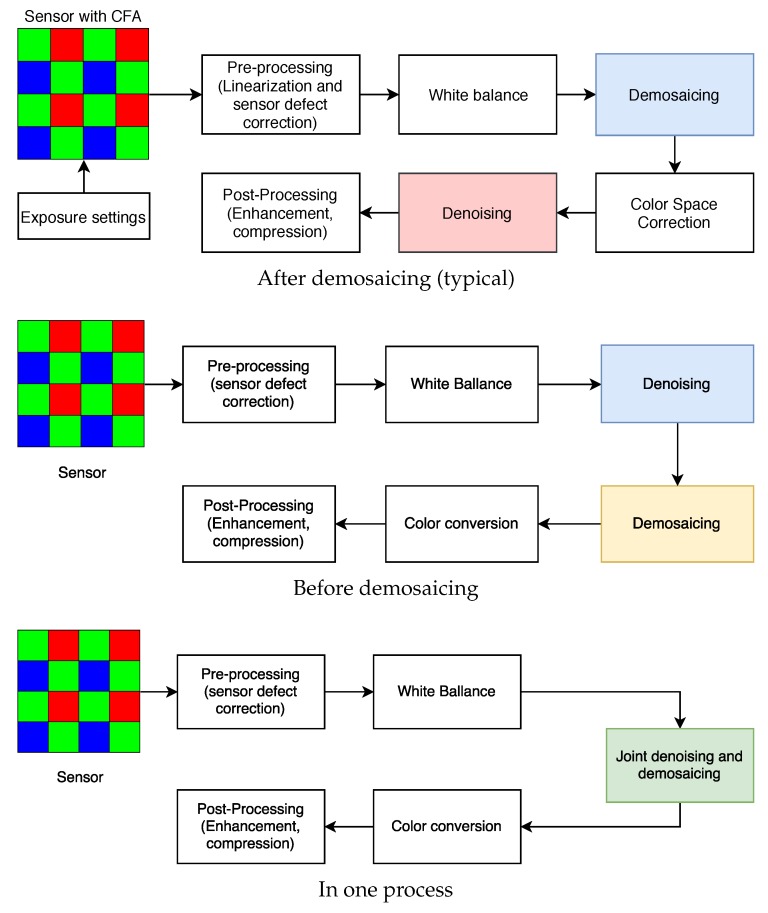
Variants of raw image processing pipelines—placement of denoising algorithms.

The process of developing digital negatives (DNG) starts with the output of the CFA sensor—a mosaic image registered in grayscale with a depth of 12 or 14 bits. The result of this process is usually a color image in sRGB or other space with a wider gamut, such as Adobe RGB. In the process of raw image developing, a number of operations should be performed, such as linearization, correction of sensor defects, white balancing, demosaicing, and denoising (Figure 2). These operations may be performed in different sequences, but noise reduction is usually one of the last steps of the raw image processing pipeline. It can be supposed that this approach is not optimal and sensor noise filtering should be done before the original distribution of sensor noise is altered by other raw image processing steps (Figure 3). However, to evaluate the real impact of choosing the filtering scheme it is necessary to assess the quality of the results.

The standard approach is still widely used, but has serious drawbacks:

- demosaicing algorithms are changing noise distribution, and introduce additional artifacts, especially in presence of impulsive noise,
- usually more data to process—three highly correlated channels (compared to one mosaic grayscale image), and
- raw image processing may cause some data loss before denoising process.

However, the classical methods are supported by the fact that most of the available noise reduction algorithms are designed to filter RGB images.

**Figure 4:**
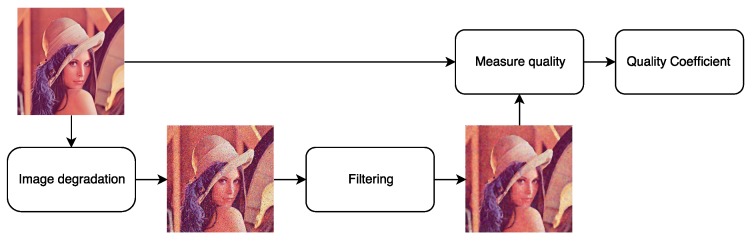
Standard approach to quality image assessment.

The process of evaluating the effectiveness of classical filtering techniques is usually based on a very simple quality evaluation model (Figure 4). This approach usually assumes the use of a standard set of test images and disrupting them with a known (usually very simple) noise model. The ideal solution would be to use real distorted images, e.g., obtained at a high ISO value and their equivalents at a minimum value. This approach was used by the authors of [16]; they use advanced techniques of matching ground truth images to their noisy counterparts. In this case, however, the noise level of ground truth images is still quite high.

## 3. Generating A Set of Test Images

In our work, we want to analyze the effectiveness of filtering methods used at different stages of image acquisition pipeline. Therefore, we have to create test images with high bit depth, suitable for evaluating the entire process of digital negative development. The proposed test image generation process uses high-quality real raw images obtained from various digital cameras.

**Figure 5:**
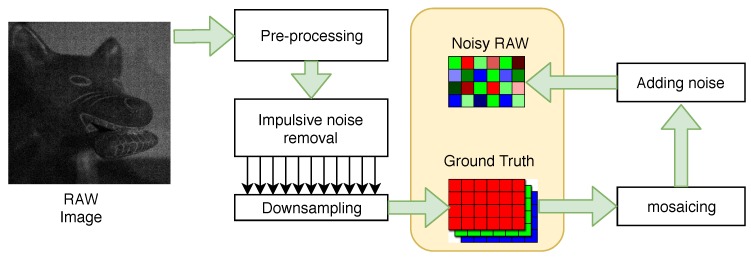
Idea of test image generation.

First the “perfect” images are created, the so-called ground truth (GT), and on their basis, images simulating the actual acquisition process are prepared (Figure 5).

### 3.1. Downsampling Real Raw Images

**Figure 6:**
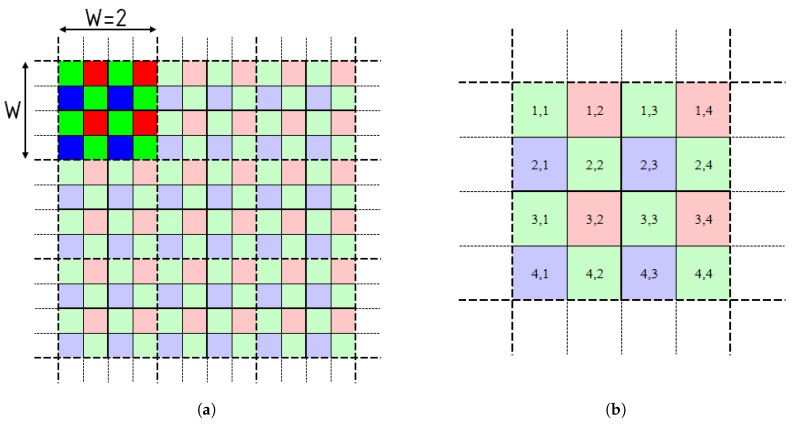
The downsampling window of size W=2 (**a**) and its indexation scheme (**b**).

To obtain high quality test images, the method presented in the paper [3] was adapted. The proposed method assumes the use of high-quality raw images, subjecting them to a significant downsampling with maximum entropy averaging. The color pixel values are determined by averaging the corresponding values from the raw image in downsampling window, an example of that window for the Bayer array is shown in Figure 6, window size W=2 means that it consists of 2 Bayer mosaic patterns in line.

**Figure 7:**
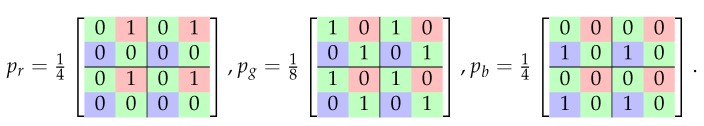
Naive averaging masks (W=2).

Averaging inside the window and assigning calculated values to a specific channel is performed using the $p_{c}$ mask, where c∈{r,g,b}. The simplest solution is to use naive averaging (Figure 7), but this approach causes color shifts in the resulting image. To compensate for the effect of shifting inside the color channels, it is necessary to choose such weights inside the masks so that their center of gravity lies in the middle of the window. Therefore, for each color channel c∈{r,g,b}, the appropriate maximization problem should be solved:

(1) $$\begin{array}{ccc} \max_{p_{c}} & -\sum_{x,y} & p_{c}\left(x,y\right)\log p_{c}\left(x,y\right), \end{array}$$

(2) $$\begin{array}{ccc} & \;\;\sum_{x,y} & p_{c}\left(x,y\right)=1, \end{array}$$

(3) $$\begin{array}{ccc} & \;\;\sum_{x,y} & x\cdot p_{c}\left(x,y\right)=W\;+0.5,\;\forall y, \end{array}$$

(4) $$\begin{array}{ccc} & \;\;\sum_{x,y} & y\cdot p_{c}\left(x,y\right)=W\;+0.5,\;\forall x, \end{array}$$

(5) $$\begin{array}{cc} & p_{c}\left(x,y\right)\geq 0,\;\forall x,y, \end{array}$$

(6) $$\begin{array}{cc} & p_{c}\left(x,y\right)=0,\;\forall \left(x,y\right)\notin C_{c}. \end{array}$$

**Figure 8:**
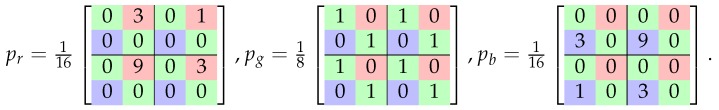
Maximum entropy averaging masks (W=2).

Constraint (2) ensures that the weights add up to one. A pair of constraints (3) and (4) ensures that the spatial center of gravity lies in the center of the window, because for the dimensions 2W×2W the center point lies in (W+0.5,W+0.5). All weights in the mask should be non-negative, which provides a restriction (5). A restriction (6) ensures that the $p_{c}$ mask weights correspond to the spatial distribution of the *c* color fields in the CFA filter, i.e., the pixel weights that do not correspond to the $C_{c}$ channel are zero. These restrictions can lead to many different weight distributions; however, the most desirable distribution is the least concentrated around some specific numerical value. The measure of this concentration is entropy, which is a function of the goal of the maximization problem posed (1). For the analyzed case of the 4×4 window (W=2), the masks that solve problems (1)–(6) take values as shown in Figure 8.

### 3.2. Impulsive Noise Problem—Raw Spatial Median Filter

Images obtained in the downsampling process described above are devoid of demosaic artifacts, in addition, the dominant sensor noise components (shot noise and read noise) are significantly reduced. However, even high-end imaging sensors may have defective pixels causing impulsive noise. This can interfere with the downsampling process by creating color artifacts in the resulting image. We can prevent this by using appropriate raw image data filtering techniques. The most effective method is to use a dark frame to detect faulty pixels and then replace them using spatial interpolation. However, if the dark frame data have not been obtained, we will use a simple modification of the Spatial Median Temporal Mean (SMTM) filter proposed in the work [17] reduced to single raw image: Raw Spatial Median Filter (RAWSM). During downsampling, for each pixel $g^{i}$ inside the 2W×2W window, the $\eta^{i}$ indicator is calculated, according to the following formula,

(7) $$\eta^{i}=\left|g^{i}-med{}_{2W\times 2W}\left\{g_{c}\right\}\right|,$$

according to the color of the CFA filter ($g^{i}\in g_{c}\in C_{c},\;c\in \left\{r,g,b\right\}$). This ensures that the evaluation of pixels of different colors is separable—whether a “blue” pixel is damaged or not can be determined from the values of the adjacent “red” pixels. The value of the $\eta^{i}$ indicator for a given pixel determines whether it is damaged or not. A defective pixel is replaced by a median of pixels belonging to the same color channel.

(8) $$g^{i}=\left\{\begin{array}{cc} m_{c}, & \eta^{i}>T\cdot \sigma \left(g_{c}\right)\\ g^{i}, & \eta^{i}\leq T\cdot \sigma \left(g_{c}\right) \end{array}\right.,$$

where $m_{c}$ is the median value used in the Equation (7), *T* is the tuning parameter of the RAWSM method, and $\sigma \left(g_{c}\right)$ is the standard deviation of the pixel value $g_{c}$. In our work, taking into account the relatively low level of impulsive noise in CMOS sensors, the suboptimal value of T=4 was selected. This filtering method was proposed for filtering during the downsampling process, but it is universal and can be used to filter raw data when processing digital negatives.

### 3.3. The Process of Preparing Ground Truth Images

Two different Canon EOS 500D and 600D digital cameras were used to obtain test high quality raw images in proprietary cr2 file format. The acquisition process was carried out at minimum sensitivity setting (ISO 100) and in good lighting conditions. The processing was carried out using the Adobe DNG converter and with the modified Matlab scripts suggested in the work [18]. The full process of sRGB ground truth image creation is described below.

- reading raw files,
- impulsive noise removal (using dark frame or with RAWSM filter),
- linearization,
- white balancing,
- maximum entropy downsampling (W=6),
- color space correction,
- brightness and contrast adjustment.

A collection of 48 virtually noise free sRGB images was obtained—25 downsampled EOS 600D photos of size 432×288, 16 bpp and 23 downsampled EOS 500D photos of size 396×264, 16 bpp. The downsampling process performed using the maximum entropy method enabled complete elimination of artifacts associated with the use of the Bayer filter matrix, and almost completely reduced the sensor noise. In addition, “perfect” images for different stages of raw image processing pipeline were prepared. Finally, four groups of ground truth test images were prepared (GT test images are available at: https://tiny.pl/t6q48):

- CFA linear - mosaiced Bayer image in linear sensor space,
- CFA sRGB - mosaiced Bayer image in sRGB color space,
- linear - RGB images in linear sensor space,
- sRGB - final sRGB images.

**Figure 9:**
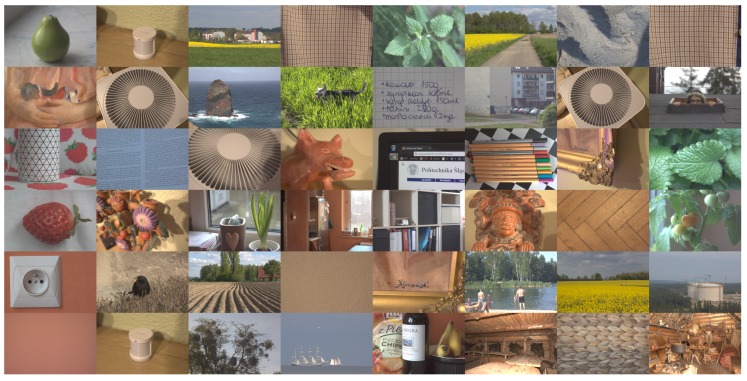
Set of test images.

**Figure 10:**
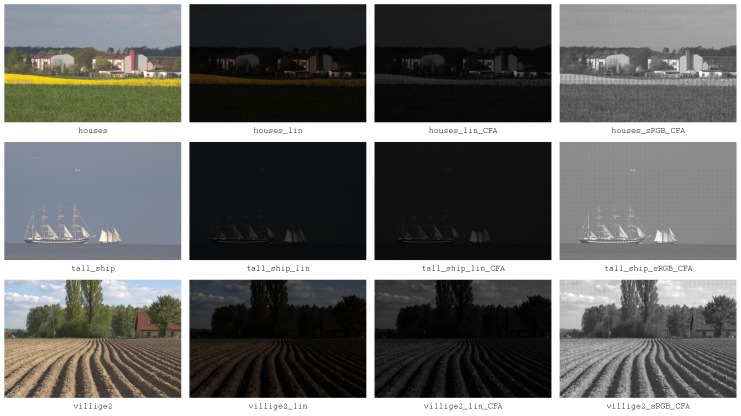
Test images for different stages of raw image processing pipeline.

Figure 9 shows set of ground truth images, whereas Figure 10 depicts examples of images in different subsets.

### 3.4. Synthetic Noise Model

The next step in the process of evaluating the quality of digital negative development is to simulate noise in a real CMOS sensor. The general model of CMOS sensor noise can be presented by additive formula:

(9) $$z\left(x\right)=y\left(x\right)+\sigma \left(y(x)\right)\cdot \xi \left(x\right),$$

where σ is a standard deviation in point *x* and ξ denotes additive white Gaussian noise (AWGN). Foi et al. [19] assume that the sensor noise consists of two independent components: signal dependent Poissonian $\eta_{p}$, and Gaussian which is independent of the signal—$\eta_{g}$:

(10) $$\sigma \left(y(x)\right)\cdot \xi \left(x\right)=\eta_{P}\left(y(x)\right)+\eta_{g}\left(x\right).$$

Variable variances of the Poissonian component $\eta_{P}$ can be determined by the linear function of the signal: *y*: $var\{\eta_{p}\left(y\left(x\right)\right)\}=ay\left(x\right)$, where *a* is a Poisson distribution parameter. The variance of Poisson noise increases with the value of the signal, while the variance of the Guassian component is constant, equal to *b*. Thus, the total variance could be written as $\sigma^{2}\left(y(x)\right)=ay\left(x\right)+b$. The general noise model can therefore be presented in a form:

(11) $$z\left(x\right)=y\left(x\right)+\sqrt{ay(x)+b}\cdot \xi \left(x\right).$$

This model contains two parameters that can be identified from imaging data obtained from real imaging sensors at various ISO values [19]. The use of a model suited to the actual device allows to obtain noise corresponding to the disturbances occurring during the actual image acquisition. This gives you the ability to test filtering algorithms for different real-world scenarios, e.g., performance depending on ISO sensitivity.

The use of such a noise model enables effective development and testing of algorithms in a near-target environment with appropriately selected interference levels (they are neither extremely low nor unnaturally high).

To more accurately reflect the real distortions in digital images, the noise model from (11) can be extended by impulsive noise component *i*:

(12) $$z\left(x\right)=y\left(x\right)+\sqrt{ay(x)+b}\cdot \xi \left(x\right)+i\left(x\right).$$

**Figure 11:**
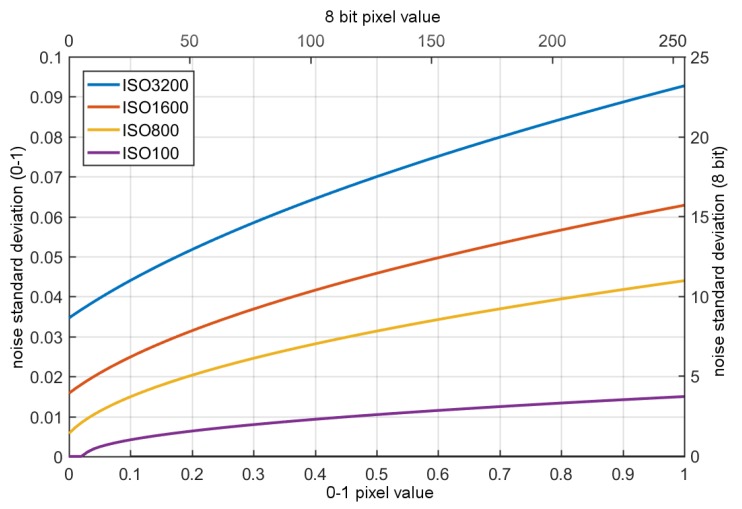
Real Gaussian–Poisson real noise parametrization for selected ISO levels.

In this model, impulse noise will be understood as a faulty pixel, often called “hot”, which can take different values, but for a given pixel the value is approximately constant. Such a noise model can be added based on dark frames obtained for various real image sensors [20]. In our case, we did not analyze impulsive noise; this is due to the fact that in the case of modern imaging sensors impulsive noise is relatively small and easy to effectively remove from the raw images, however impulsive noise data obtained from real camera were also included in our data set (The noisy images are available at: https://tiny.pl/t6xtj). The model described by Equation (11) corresponds to the physical phenomena occurring in the imaging sensors causing the dominant types of noise: shot and read out noise. However, it is necessary to determine realistic values of model parameters; in our case, we used the procedure proposed by Foi et al. in the works [19]. In our study, the noise model parameters were determined for Canon EOS 500D for four different ISO values (100, 800, 1600, and 3200), the obtained parameter values are shown in Figure 11. This model can be used to model data for both monochrome sensors and those using a Bayer filter, but must be used for raw data, as the noise/signal variance dependence has been determined for such data.

## 4. Experiment

### 4.1. The Assumptions of the Experiment and the Input Data

During many years of work on image filtering, many noise reduction algorithms have been developed, some of them also have versions adapted to raw data processing. For our research we chose well known and effective algorithms using the idea of non-local means (NLM) [21] and BM3D [8]. Both algorithms are often used as a reference in filtering efficiency testing, and BM3D is still one of the most effective algorithms for filtering Gaussian-like noise. There are also many modifications to these algorithms that significantly improve their speed. Although both NLM and BM3D have been adapted to work with CFA data, we also decided to test the solution presented in the work [22]—pseudo four-channel filtering technique (P4Ch). This method is based on decomposition of CFA image into four smaller sub-images (pseudo-channels) corresponding to pixel positions in Bayer pattern. Then the channels are subjected to PCA analysis, so obtained channels can be filtered using any technique designed for grayscale images. In the last step, we perform reverse transformations to obtain a filtered CFA image. It is possible to create four different variants of P4Ch sub images (*RGGB*, *GRBG*, *BGGR*, and *GBRG*), so the P4Ch filtering operation is repeated four times and the final CFA result is obtained by averaging the values obtained for all variants.

Another problem is the choice of demosaicing algorithms, in our case, we decided to choose two algorithms: the Adams Hamilton algorithm (AH) [23] and Self-similarity driven demosaicing (SSDD) [24]. The first one is quite popular because of its relative simplicity and high efficiency, while the second one was created as a development of the idea contained in the NLM algorithm, giving excellent results, which is, however, paid for by the high computational complexity.

During our research, we tested the operation of various filtering and demosaicing scenarios. We have performed tests for different levels of noise for all images in our image data set. In addition, each image was corrupted independently ten times to reduce the uncertainty of the result caused by the use of a pseudo-random number generator. During the research, we tested different combinations of filtering and demosaicing algorithms; in the final comparison we used the following methods,

- classical color image denoising filters:
  – Non Local Means (NLM) [21],
  – Color Block-Matching and 3D Filtering (CBM3D) [8],
- direct raw denoising [25]:
  – CFA NLM,
  – CFA BM3D,
- pseudo 4-channel filtering technique [22] (P4Ch)
  – P4Ch NLM,
  – P4Ch BM3D,
- demosaicing techniques:
  – Adams Hamilton (AH) [23],
  – Self-similarity-driven demosaicing (SSDD) [24].

Noisy raw images are the starting point for our tests, and for their processing, it is necessary to use an appropriate combination of filtering and demosaicing; the appropriate processing sequences are shown in Table 1. Due to the specificity of the tested algorithms, we divided them into three groups: standard filters, pseudo-four channel filters (P4Ch), and direct CFA filters.

During our experiment, numerous simulations were carried out and all the filter combinations shown in Table 1 were tested, so finally we have obtained:

- 48 ground truth images,
- 4 noise levels,
- 10 noise process realizations,
- 14 filtering scenarios,
- 1920 test input images, and
- 26,880 filtering results.

### 4.2. Results

**Figure 12:**
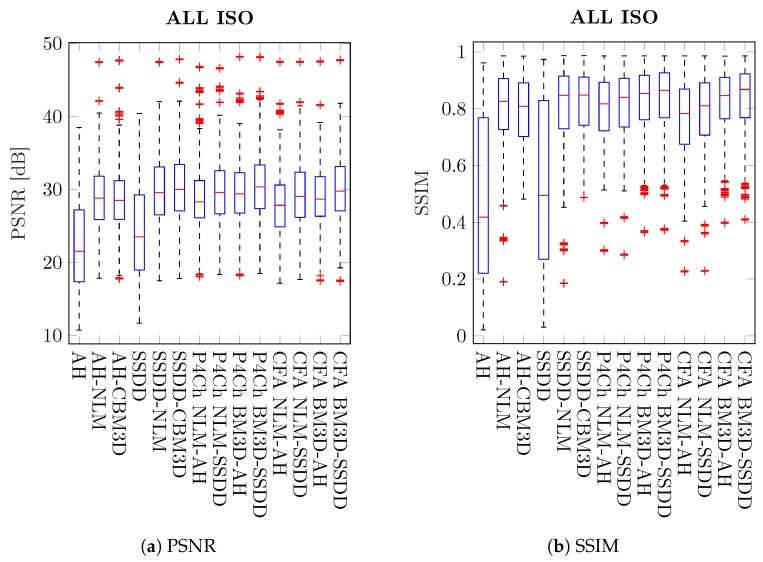
Box plots of quality coefficients for all images and noise levels.

Table 2 and Table 3 show the mean results of peak signal to noise ratio (PSNR) and structural similarity index (SSIM) as well as standard deviations for the whole image test set. The results in the tables are color-coded and grouped according to the classification used in Table 1, the best results are highlighted in green. The results obtained for the whole data set, i.e., for all images and for all ISO sensitivities (noise levels) were visualized on boxplots (Figure 12).

**Figure 13:**
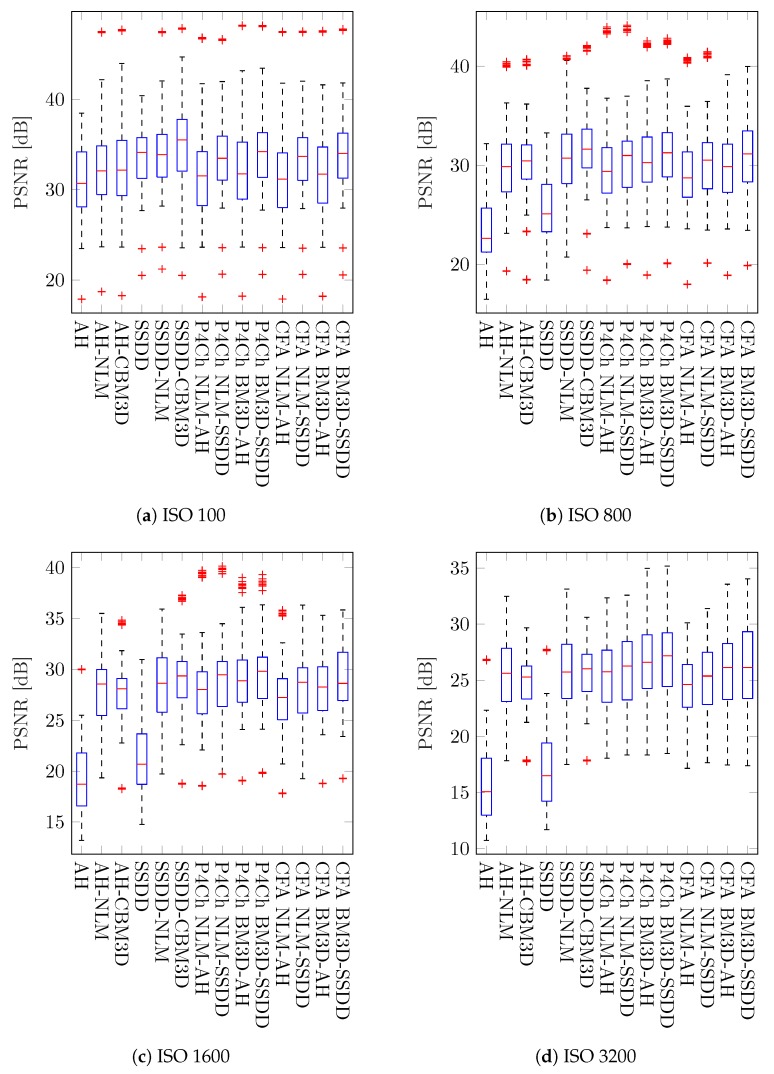
Box plots of PSNR values for all images depending on the noise level.

From these results, it is difficult to draw general conclusions about the whole set of images and all levels of noise. Average results for the best classical methods are similar to those achieved for filters using raw data from the Bayer matrix. It is therefore also not possible to answer the general question posed at the beginning of this work: which of the processing schemes will give the best results. However, by analyzing the results for different ISO sensitivities, we see that as the noise level increases, the more advanced raw filtering methods gain a noticeable advantage, which is also visible on the boxplots presented in Figure 13.

Another method of analysis of the tested results may be a kind of rank assessment—grouping the compared techniques by the number of files for which a given method wins. Table 4 groups the results obtained for different ISO sensitivities using the PSNR indicator.

The results confirm the advantage of methods based on direct raw data filtering over classical methods for higher noise levels.

According to this comparison, for high noise levels (ISO 1600 and 3200), the best results are obtained for the most advanced P4Ch BM3D filtering technique using SSDD interpolation. Minimally worse results are obtained much faster, and thus are more useful, by the Adams–Hamilton demosaic algorithm. What is more, when on both sides of the comparison we place classical methods and methods performing filtering before the interpolation stage, the advantage of the latter will be even more visible.

**Figure 14:**
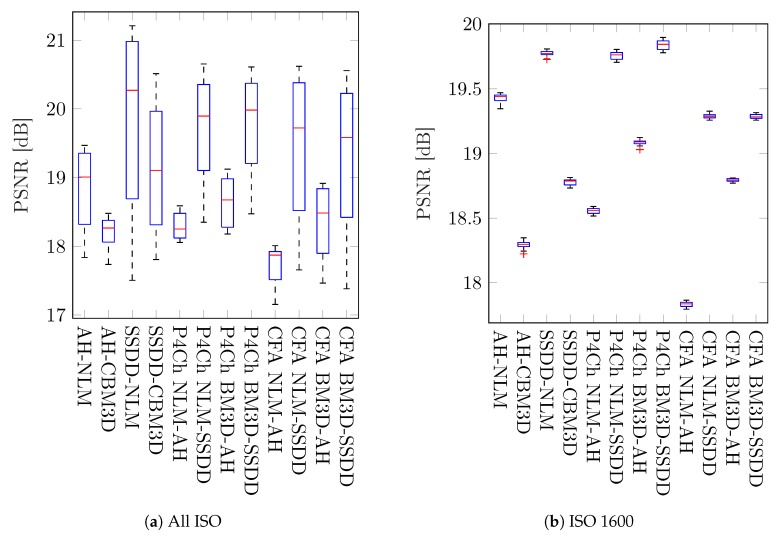
Box plots of PSNR coefficient for the *dog* test image.

**Figure 15:**
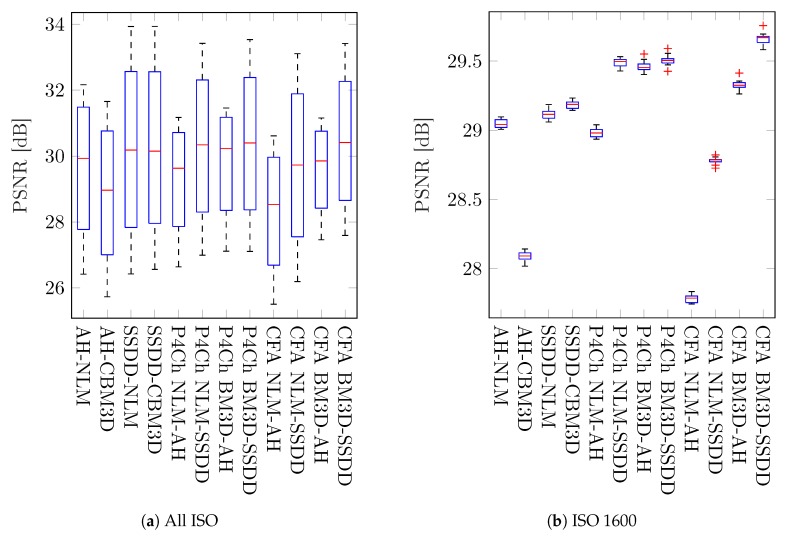
Box plots of PSNR coefficient for the *bird* test image.

**Figure 16:**
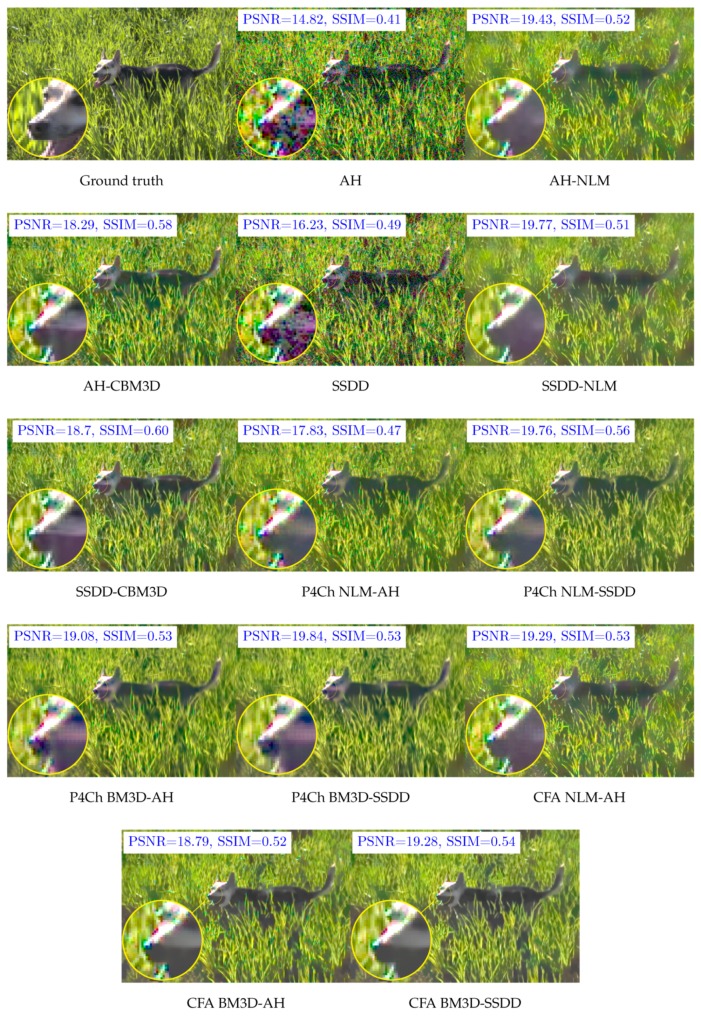
Filtering results for *dog* test image—ISO 1600.

**Figure 17:**
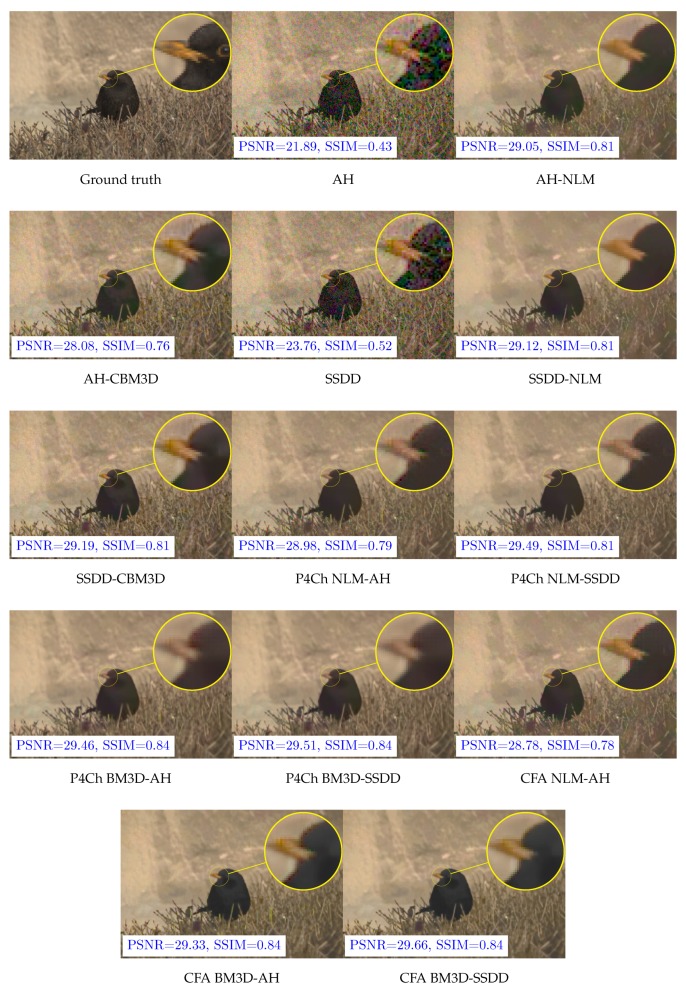
Filtering results for *bird* test image—ISO 1600.

We also decided to look at the results for individual files, we chose two images *dog* and *bird*, the first one being quite a challenge for filtering and demosaicing algorithms—its results often represent outliers, while the second one gives results typical for our data set. The relevant PSNR box plots obtained for these images for all ISO sensitivities and ISO 1600 are shown in Figure 14 and Figure 15 (for better readability, these graphs skip data for the demosaic operation itself without filtering). Examples of visual filtering results for noise corresponding to ISO 1600 are shown in Figure 16 and Figure 17. Analyzing the numerical results and images obtained for the test image “dog” we can assume that in this case, the demosaicing process is more difficult than filtering, which is indicated, among other things, by the great advantage of methods using an advanced SSDD algorithm. Nevertheless, in this case, for higher noise values, better results are obtained by placing the filtering process before interpolation. Note that, taking into account the structural quality coefficient (SSIM), the benefits of using direct raw image filtering methods may be greater than for the PSNR indicator.

Despite a relatively small set of test images, we tried to include data with great diversity in it. The test set included images with a large number of details, textural data, and images containing uniform surfaces. The images are characterized by different colors in terms of both color temperature and saturation. In our simulations, we tried to recreate as faithfully as possible the natural process of noise generation during image acquisition; additionally, each image was disturbed by 10 modeled noise realizations. As can be seen on the boxplots in Figure 14b and Figure 15b, the influence of randomness on the interference on the variability of obtained results is relatively small.

Analyzing the obtained results, especially the boxplots (Figure 12 and Figure 13), it can be seen that the obtained results have a large range of variability for different images and levels of noise and it is difficult to assess whether they are statistically significant. To assess the statistical significance of the results obtained, a two-sample t-test was carried out.

For simplicity, we have divided our algorithms into two classes: classical algorithms (demosaic, then denoise) and algorithms dedicated to CFA data filtering (denoise, then demosaic). We have compared the results for the best classical and CFA filtering methods, grouping the results according to ISO values. In all comparisons, we assumed the null hypothesis with equal average quality coefficients (PSNR or SSIM) with a significance level of 5%. The alternative hypothesis, on the other hand, assumed that the average quality index for the winning filter is higher than the second one. Moreover, we assumed that the compared sets may have different variances.

The results obtained are presented in the form of box charts for the methods compared, in each case the better method is placed in the chart on the left. The value of h=1 means that the obtained result for our data is statistically significant (i.e., we reject the null hypothesis).

**Figure 18:**
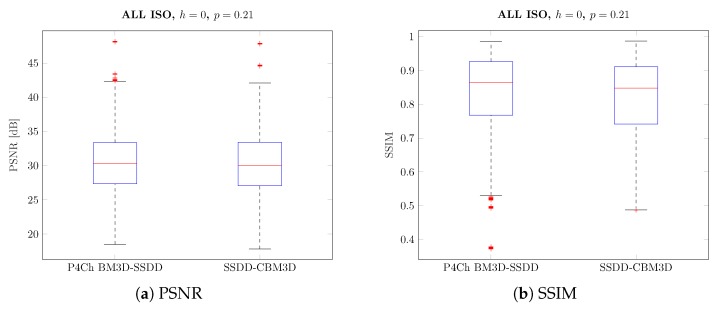
Pairwise comparison of different filtering approaches—full image dataset.

At the beginning, we compared the results for the whole test set—all pictures and all ISO sensitivities, the results of the comparison are shown in Figure 18. As it could be assumed after analyzing the data in Table 2 and Table 3, the test confirms that the difference between these results is not statistically significant (h=0, *p*-value = 0.21).

**Figure 19:**
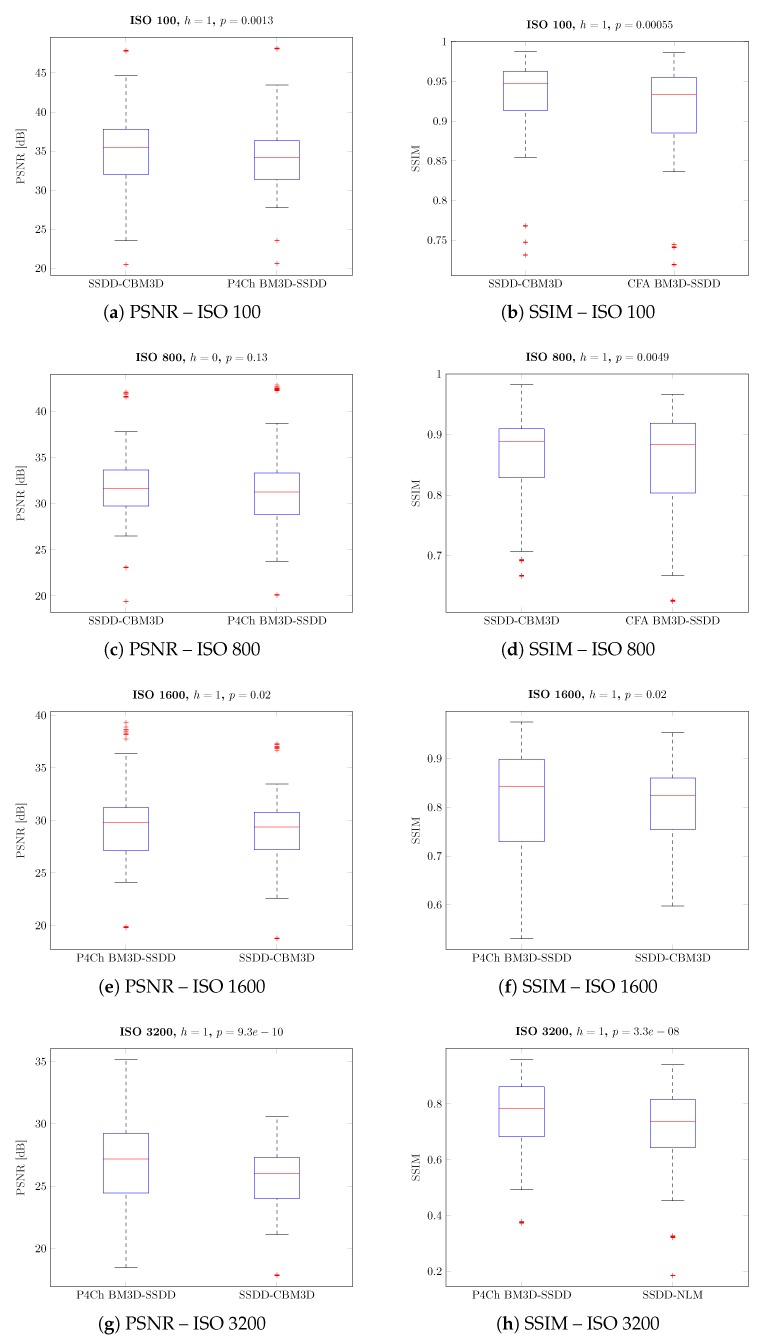
Pairwise comparison of different filtering approaches - grouped by ISO level.

Subsequent tests were carried out for individual noise levels (ISO values)—the results obtained are shown in Figure 19, as can be seen in most cases, the differences obtained are statistically significant. As previously observed for small ISO values, better results were obtained for classical filtering methods, while for larger noise levels, CFA data filtering seems to be a better solution.

## 5. Conclusions

The presented paper analyzes the influence of location of filtering algorithms in the whole pipeline digital negative development on the final image quality. A high-quality image data set has been prepared, which, according to the authors, made it possible to carry out tests in conditions close to real life. Our results show that the best approach to filtering CFA images for all cases can not be clearly identified. In general it seems that the traditional approach gives better results for images with relatively small amount of noise, whereas with the increase of noise, dedicated raw filtering techniques give better and better results. However, it should be noted that we have obtained results for a limited set of images, which may limit confidence in the results. Appropriate diversity of images and a clear advantage of dedicated RAW methods allow us to believe that these results can be generalized. In general, it can be assumed that the predominance of traditional methods for small noise levels is small enough to recommend the use of more advanced methods in all cases. The obtained results can help in choosing the best approach to the process of processing raw images—directly in digital cameras, as well as in post-processing software. It seems that for low light conditions it is crucial to carry out noise reduction before the CFA data interpolation process.

## Figures and Tables

**Table 1:** Filtering and demosaicing scenarios used in our experiments divided into three groups.

No.	Standard Filters	No.	P4Ch CFA Approach	No.	Direct CFA Filters
1	AH	7	P4Ch NLM → AH	11	CFA NLM → AH
2	AH → NLM	8	P4Ch NLM → SSDD	12	CFA NLM → SSDD
3	AH → CBM3D	9	P4Ch BM3D → AH	13	CFA BM3D → AH
4	SSDD	10	P4Ch BM3D → SSDD	14	CFA BM3D → SSDD
5	SSDD → NLM				
6	SSDD → CBM3D				

**Table 2:** Peak signal to noise ratio (PSNR) average results—all files.

Method	All ISO		ISO100		ISO800		ISO1600		ISO3200	
	PSNR	σ	PSNR	σ	PSNR	σ	PSNR	σ	PSNR	σ
AH	22.26	6.67	30.75	4.19	23.30	3.31	19.30	3.61	15.68	3.40
AH → NLM	28.91	4.52	32.15	4.77	29.88	3.65	28.10	3.37	25.50	3.29
AH → CBM3D	28.84	4.54	32.42	5.19	30.12	3.52	27.88	2.72	24.95	2.27
SSDD	24.22	7.01	33.30	3.92	25.55	3.18	21.10	3.55	16.95	3.40
SSDD → NLM	29.69	4.84	33.82	4.37	30.60	3.83	28.53	3.66	25.79	3.51
SSDD → CBM3D	30.28	4.86	34.70	4.73	31.50	3.64	29.11	3.09	25.81	2.60
P4Ch NLM → AH	28.75	4.46	31.59	4.81	29.69	3.89	28.12	3.49	25.59	3.15
P4Ch NLM → SSDD	29.74	4.68	33.43	4.38	30.64	3.86	28.82	3.62	26.09	3.43
P4Ch BM3D → AH	29.58	4.45	31.98	5.02	30.45	3.88	29.07	3.52	26.81	3.51
P4Ch BM3D → SSDD	30.41	4.65	33.79	4.60	31.23	3.86	29.55	3.60	27.05	3.65
CFA NLM → AH	28.03	4.50	31.36	4.92	28.98	3.72	27.18	3.22	24.61	2.84
CFA NLM → SSDD	29.28	4.70	33.40	4.41	30.21	3.66	28.15	3.35	25.36	3.12
CFA BM3D → AH	29.01	4.46	31.80	4.97	29.90	3.65	28.37	3.28	25.99	3.57
CFA BM3D → SSDD	29.99	4.74	33.72	4.51	30.89	3.72	29.01	3.48	26.32	3.83

**Table 3:** Structural similarity index (SSIM) average results—all files.

Method	All ISO		ISO100		ISO800		ISO1600		ISO3200	
	SSIM	σ	SSIM	σ	SSIM	σ	SSIM	σ	SSIM	σ
AH	0.477	0.288	0.873	0.065	0.500	0.166	0.332	0.171	0.201	0.132
AH → NLM	0.799	0.133	0.902	0.067	0.827	0.095	0.773	0.121	0.694	0.141
AH → CBM3D	0.793	0.120	0.912	0.066	0.844	0.067	0.766	0.067	0.648	0.077
SSDD	0.530	0.288	0.907	0.051	0.580	0.162	0.394	0.181	0.237	0.147
SSDD → NLM	0.809	0.139	0.912	0.063	0.835	0.103	0.782	0.133	0.709	0.150
SSDD → CBM3D	0.825	0.112	0.930	0.056	0.868	0.069	0.808	0.075	0.694	0.079
P4Ch NLM → AH	0.795	0.121	0.897	0.069	0.829	0.082	0.773	0.095	0.679	0.113
P4Ch NLM → SSDD	0.812	0.121	0.912	0.061	0.842	0.084	0.790	0.100	0.705	0.122
P4Ch BM3D → AH	0.826	0.114	0.899	0.071	0.845	0.089	0.807	0.106	0.753	0.130
P4Ch BM3D → SSDD	0.835	0.117	0.913	0.063	0.853	0.091	0.814	0.109	0.759	0.134
CFA NLM → AH	0.763	0.137	0.890	0.073	0.804	0.089	0.734	0.105	0.625	0.114
CFA NLM → SSDD	0.789	0.134	0.909	0.062	0.825	0.090	0.761	0.111	0.663	0.123
CFA BM3D → AH	0.823	0.111	0.901	0.071	0.849	0.083	0.804	0.096	0.739	0.118
CFA BM3D → SSDD	0.835	0.113	0.917	0.061	0.858	0.085	0.814	0.101	0.750	0.124

**Table 4:** Number of “wins” for individual filtering schemes according to the achieved value of PSNR coefficient.

No.	Filter	All ISO	100	800	1600	3200
1	AH	0	0	0	0	0
2	AH → NLM	0	1	1	0	0
3	AH → CBM3D	0	2	0	0	1
4	SSDD	0	0	0	0	0
5	SSDD → NLM	4	5	8	2	0
6	SSDD → CBM3D	21	37	26	14	6
7	P4Ch NLM → AH	0	0	0	0	0
8	P4Ch NLM → SSDD	2	0	1	2	2
9	P4Ch BM3D → AH	2	1	0	2	2
10	P4Ch BM3D → SSDD	15	2	9	20	26
11	CFA NLM → AH	0	0	0	0	0
12	CFA NLM → SSDD	0	0	0	0	0
13	CFA BM3D → AH	0	0	0	0	1
14	CFA BM3D → SSDD	6	0	3	8	10
